# Genetic variability of the U5 and downstream sequence of major HIV-1 subtypes and circulating recombinant forms

**DOI:** 10.1038/s41598-020-70083-1

**Published:** 2020-08-06

**Authors:** Christelle Mbondji-Wonje, Ming Dong, Jiangqin Zhao, Xue Wang, Aubin Nanfack, Viswanath Ragupathy, Ana M. Sanchez, Thomas N. Denny, Indira Hewlett

**Affiliations:** 1 Laboratory of Molecular Virology, Division of Emerging and Transfusion Transmitted Diseases, Center for Biologics Evaluation and Research, Food and Drug Administration, Silver Spring, MD USA; 2 U.S. Military HIV Research Program, Walter Reed Army Institute of Research, Silver Spring, MD USA; 3 Henry M. Jackson Foundation for the Advancement of Military Medicine, Bethesda, MD USA; 4 Chantal BIYA International Reference Centre for Research on HIV/AIDS Prevention and Management (CIRCB), Yaoundé, Cameroon; 5 Duke Human Vaccine Institute, Department of Medicine, Duke University Medical Center, Durham, NC 27710 USA; 6 Present Address: Laboratory of Molecular Virology, Division of Emerging and Transfusion Transmitted Diseases, Center for Biologics Evaluation and Research, Food and Drug Administration, Building 72, Rm 4230 10903 New Hampshire Avenue, Silver Spring, MD 20993 USA

**Keywords:** Genetics, Genome, Structural variation, Diseases, Infectious diseases, HIV infections

## Abstract

The critical role of the regulatory elements at the 5′ end of the HIV-1 genome in controlling the life cycle of HIV-1 indicates that this region significantly influences virus fitness and its biological properties. In this study, we performed a detailed characterization of strain-specific variability of sequences from the U5 to upstream of the *gag* gene start codon of diverse HIV-1 strains by using next-generation sequencing (NGS) techniques. Overall, we found that this region of the HIV-1 genome displayed a low degree of intra-strain variability. On the other hand, inter-strain variability was found to be as high as that reported for *gag* and *env* genes (13–17%). We observed strain-specific single point and clustered mutations in the U5, PBS, and *gag* leader sequences (GLS), generating potential strain-specific transcription factor binding sites (TFBS). Using an infrared gel shift assay, we demonstrated the presence of potential TFBS such as E-box in CRF22_01A, and Stat 6 in subtypes A and G, as well as in their related CRFs. The strain-specific variation found in the sequence corresponding at the RNA level to functional domains of the 5ʹ UTR, could also potentially impact the secondary/tertiary structural rearrangement of this region. Thus, the variability observed in this 5′ end of the genomic region of divergent HIV-1 strains strongly suggests that functions of this region might be affected in a strain-specific manner. Our findings provide new insights into DNA–protein interactions that regulate HIV-1 replication and the influence of strain characterization on the biology of HIV-1 infection.

## Introduction

Human immunodeficiency virus type 1 (HIV-1) is characterized by extensive genetic diversity. HIV-1 group M, which accounts for the majority of infections worldwide, has been subdivided into at least nine genetically distinct subtypes (A–D, F–H, J, and K), a few sub-subtypes (A1-A3, F1, F2), and numerous circulating and unique recombinant forms (CRFs and URFs respectively). HIV-1 diversity is featured by high mutagenesis rates and recombination events occurring during the life cycle of the virus. Nucleotide changes generated from these mechanisms affect the entire viral genome, including its highly structured 5′ end region. Indeed, the HIV-1 genome variability was found to be as much as 23% in the 5′LTR-U3 region, about 10% in the *gag/pol* genes, and more than 30% in the *env* gene, with overall differences observed primarily between intra and inter-subtype^1,2^. Previous studies have revealed that strain differences may influence transmission, replication, and virulence of HIV-1. It was reported that CRF01_AE has a higher rate of sexual transmission than subtype B^3^, while this rate was higher in subtype A compared to D^4^. Subtype A was also shown to have a lower replication rate than subtype C^5^, and a lower rate of disease progression than its related CRFs and subtype D^4^. Association of these differences with the variability of specific genomic regions remains to be clarified. Nevertheless, it was suggested that the *gag*-protease region is a major determinant of disease progression differences among HIV-1 subtypes^6^, whereas differences in *pol* gene might be associated with differences in replication capacity as well as disease progression^7^. Further, some configurations in the 5′LTR were correlated with clinical disease severity and viral reactivation^8,9^, while mutations associated with viral fitness recovery were shown to be located within the 5′ untranslated region (5ʹ UTR)^10^.

**Figure 1:**
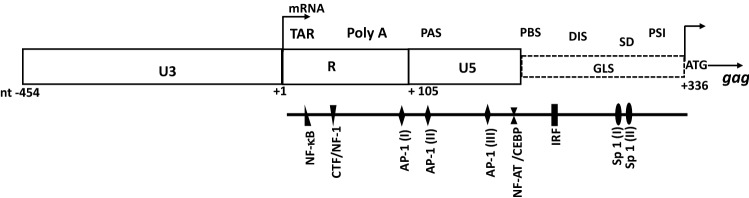
Schematic representation of the 5′LTR (U3, R, and U5 regions) and the *gag* leader sequence (GLS). ATG represents the start codon of the *gag* gene. Known TFBS sequences downstream of the transcription initiation site + 1 (U3–R junction) are depicted. Location of functional domains such as TAR (trans-activation responsive element), Poly A (polyadenylation hairpin), PAS (polyadenylation signal), PBS (Primer Binding Site), DIS (Dimer Initiation Signal), SD (major splice Donor Site) and PSI (Packaging Signal) reported on the transcribed untranslated region known as the 5′ UTR, are also depicted. (Adapted from references^11,12,14^).

As previously demonstrated, activation of the HIV-1 genome requires the binding of host cell transcription factors to regulatory elements located at its 5′end. Sequence motifs upstream and downstream of the R region of the 5′LTR have been demonstrated to interact extensively with host cell factors^11,12^. The 5′ end of the HIV-1 genome encompasses nucleotide sequences corresponding at the RNA level, to the non-coding 5′ UTR. This sequence of approximately 400 bases includes the R, U5, and primer binding site (PBS) region, as well as the *gag* leader sequence (GLS) positioned upstream of the *gag* gene initiation codon^11,13^ (Fig. 1). In this sequence, several regulatory elements were shown to bind distinct families of transcription factors such as CTF/NF-I, NF-kB, C/EBP, AP-1, NF-AT, SP-1, and IRF^12,14^ (Fig. 1).

At the RNA level, the 5′ UTR was described as a complex set of hairpin structures that include several functional domains (Fig. 1). The R region contains the trans-activation responsive (TAR) element that mediates activation of transcription through its binding to the Tat viral protein and the poly A hairpin. The poly A hairpin involved in the tight control of polyadenylation is suppressed at the 5′ end, while the sequence at the 3′ end is active^15^.

The adjoining nucleotide sequence includes the U5 and the PBS region that have been shown to play a critical role in initiating reverse transcription, notably through the annealing of a cellular tRNA-lys^3^ molecule to the viral PBS^16,17^. Further downstream, the dimer initiation signal (DIS), the major splice-donor site (SD), and the packaging signal (PSI) involved in the dimerization, splicing, and packaging of the viral RNA respectively, are contained within the GLS^11,18–20^. The stability of the conformational secondary structure characteristic of the 5′ UTR was shown to be necessary for its optimal functionality^17,21^. Hence, the 5′ UTR is a crucial modulator of various processes of the replication of HIV-1, and variability in this region may contribute to the differences observed in the biological properties of HIV-1 variants.

To date, most reports on regulatory elements that bind transcription factors at the 5′ end of the HIV-1 genome have focused on the sequence upstream of the U5 region. Although the nucleotide sequence reverse-transcribed into the 5′ UTR has been considered as the most conserved region of the viral genome^22^, a few studies have reported strain-specific variation in the R and U5-PBS region^1,23–26^. Yet, detailed strain-specific variability of this region remains poorly documented. With its ongoing genetic evolution of HIV-1, characterizing the intra-and inter variability of the 5′ UTR from diverse HIV-1 strains, including recently reported CRFs such as CRF22_01A1, may be warranted. Here, we studied the inter and intra-strain variability of the U5 region and downstream sequence at the 5′ end genomic region of various HIV-1 subtypes and CRFs. Characterizing the variability of the 5′ UTR is important as detailed information of strain-specific differences in this region of the viral genome may provide new insights into mechanisms that promote strain-specific fitness and pathogenicity. This information could contribute to a better understanding of the differences in the global distribution and expansion of specific HIV-1 subtypes/CRFs.

## Result

In this study, we analyzed the genetic variability of the U5 and downstream sequence using 80 contigs assigned to HIV-1 subtype B (n = 20), subtype C (n = 8), D (n = 10), subtype F2 (n = 5), subtype G (n = 3) subtype A1 (n = 10); CRF02_AG (n = 9), CRF01_AE (n = 10) and CRF12_BF (n = 5). The five-sequences assigned to be CRF12_BF were additional contigs retrieved from HIV-1 subtype B specimens. Also, one additional contig from a CRF02_AG isolate was subtyped as HIV-1 F2, and all the sequences retrieved from HIV-1 CRF22_01A1 isolates (n = 5) were assigned to be CRF01_AE.

### Nucleotide variability in the 5′ UTR region

Compared to HBX2, the nucleotide sequence identity of the U5 region was found to be less than 74% in CRF specimens, around 78% in subtype G, and ranged from 82% in subtype A1 to more than 93% in the other HIV-1 strains analyzed. The overall inter-strain variability of the U5 and PBS region was about 17% on average, while intra-strain variability was low with a percentage of identity higher than 93% for all HIV-1 strains studied but, subtype A1 for which the intra-strain diversity was about 18%. When compared with each other, we found that the U5-PBS region of CRF22_01A1 isolates shared a nucleotide identity of about 96% with CRF01_AE, 87% with CRF02_AG and less than 75% with isolates belonging to strains other than subtypes A1 and G. Further, sequences from subtypes B, C, D, and F2 displayed more than 92% nucleotide identity in this 5′ UTR region. In comparison with HXB2, we observed that the GLS region of subtype B isolates shared about 94% of nucleotide identity, while for the non-B strains, it was less than 85%. Nucleotide sequences constitutive of the GLS region were also well conserved between sequences from the same strain with an identity of 93% on average. For this region, inter-strain variability was up to 20% between subtype D and G and as low as 5% between CRF02_AG and CRF22_01A1.

### Strain differential nucleotide change in the U5 sequence

**Figure 2:**
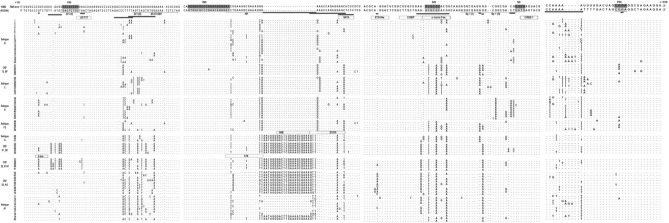
Alignment of the U5 region and downstream sequence of various HIV-1 subtypes and circulating recombinant forms. Nucleotide positions are relative to HXB2 (accession number K03455). Identity is indicated by dots; insertions/deletions are indicated by dashes. Several landmarks such as PAS (polyadenylation signal), PBS (Primer Binding Site), DIS (Dimer Initiation Signal), SD (major splice Donor Site), and PSI (Packaging Signal) motifs of the corresponding 5′ UTR sequence are depicted. Previously reported transcription factors are underlined^11,12,14,27^. Strain-specific variability is represented as hatched rectangles below the sequence; strain-specific transcription factors predicted and identified by gel shift are indicated as dash and solid rectangles, respectively.

Nucleotides from + 107 to + 140, which include sequence transcribed into the PAS motif (nt + 123 to + 130) was conserved across HIV-1 strains (Fig. 2). However, we observed some strain-specific variation within its neighboring nucleotides. Thus, the TTGTGT sequence (nt + 117 to + 122) at the 5′ end of the sequence corresponding to the PAS, was converted into a TGTTAG motif in CRF01_AE specimens as previously reported^24,26^, and also in about 40% of the HIV-1 CRF22_01A1. In the remaining 60% of HIV-1 CRF22_01A1 sequences, we observed a TGAiTTA(G/A) consensus (Fig. 2). These mutations lead to a noticeable variation of the canonical sequence of the AP-1(II) reported at this position^27^.

**Figure 3:**
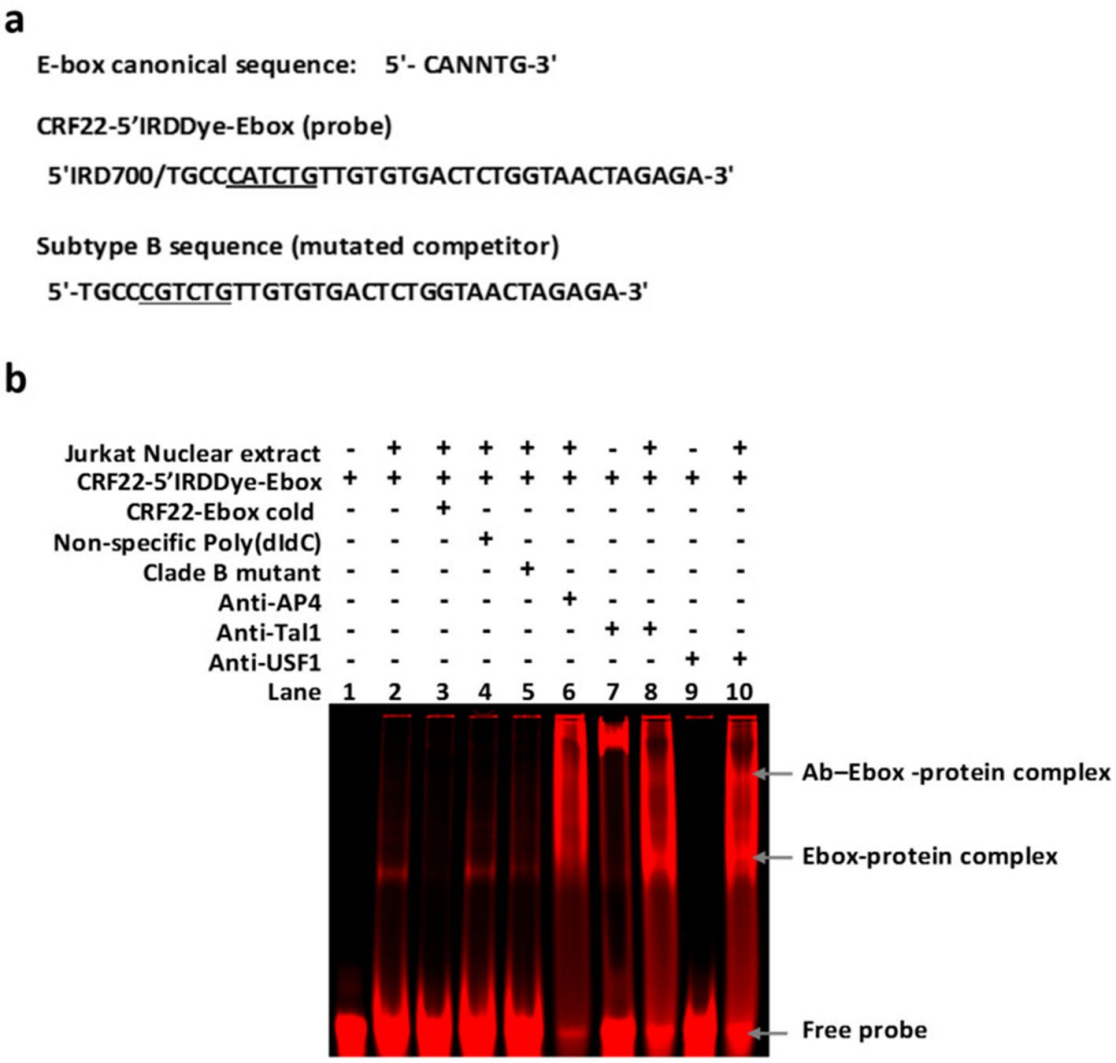
Strain-specific E-box motif binding site for Tal1 and USF1 in the U5 region of CRF22_01A1 HIV-1 strain. (**a**) E-box canonical and sequence from nucleotide + 107 to + 140 in the U5 region. The CRF22-5′IRDDye-Ebox, which includes the perfect match of the E-box canonical motif (underlined) found in the CRF22_01A1, was used as a probe. Its counterpart from clade B was used as a mutated competitor. (**b**) The CRF22-5′IRDDye-Ebox oligonucleotide probe was incubated without (lane 1) or with Jurkat nuclear extract showing a DNA–protein complex (lane 2). The specificity of the Ebox-bHLH proteins complex was suggested by the addition of a 100-fold excess of unlabeled CRF22_01A1 sequence (lane 3), a non-specific poly(dI-dC) competitor (lane 4) or a mutated competitor (lane 5). Additionally, we observed a supershift in the E-box-bHLH complex in the presence of antibodies against TAL-1 (lane 8) and USF1 (lane 10). No supershift was seen in the presence of specific antibodies, such as anti-AP4 (lane 6). Also, there was no shift in the presence of the probe and antibodies alone (lanes 7 and 9).

The HXB2 CGTCTG motif (nt + 111 to + 116) was changed to a CATCTG variant, matching the canonical E-box motif (CANNTG) in all the CRF22_01A1, in 50% of the subtype D, and 40% of the CRF01_AE specimens (Fig. 2). The ConTra v3 software predicted this variant as a binding site for bHLH proteins such as Tal1, E47, AP-4, USF1, and E47. To investigate the binding of some of these proteins to the potential E-box, we performed a supershift analysis using the CRF22_01A1 sequence infrared labeled as a probe and its counterpart in subtype B a mutated competitor (Fig. 3a).

A specific DNA–protein complex was observed with the nuclear extract of Jurkat cells (Fig. 3b lane 2). The E-box specificity of the bands was shown by a non-disruption of the DNA–protein complex with a non-specific oligonucleotide (Fig. 3b lane 4) or with the mutated competitor (Fig. 3b lane 5). On the other hand, the addition of an excess molar concentration of the cold probe as a specific competitor completely abrogated the DNA-Ebox complex (Fig. 3b lane 3). We observed a weak supershift of the E-box-bHLH proteins complex in the presence of anti-Tal1 (Fig. 3b lane 8) and a brighter band with anti-USF1 (Fig. 3b lane 10) antibodies. No supershift of the DNA–protein complex was observed with anti-AP4 (Fig. 3b lane 6), and no shift was noted between selected antibodies and the designed probes (Fig. 3b lane 7 and 9 respectively). These results indicated that the U5 region of CRF22_01A1 might harbor an E-box that binds USF-1 and, to a lower extent, Tal1 transcription factors. The specificity of this binding was emphasized with the absence of a supershift in the presence of antibodies against c-Myc, TCF3, GATA related proteins, or IgG observed in the continuation gel run in parallel (supplementary information S1 Fig. 3b).

At the 3′ end of the PAS motif, the overlapping GTAACTA (nt + 130 to + 136) sequence was conserved in about 85% of the sequences analyzed. This sequence resembling the reported consensus motif for TCF/LEF-1 factor^28^, was observed in all HIV-1 strains studied except CRF22_01A1 specimens where the GTTACTA variant was prevalent. Further downstream (nt + 148 to + 175), the variability of the nucleotide sequence reported to encompass recognition sites for AP-1 (III), C/EBP, and NF-AT proteins^27,29,30^, was fairly high in our study. The GACCCT sequence (nt + 148 to + 153) mainly found in Subtype B and C isolates, was changed to a GACCTC variant in subtype D (80%), and to a GACCA(C/T) consensus in all CRFs tested as well as in a majority of sequences from subtypes G, A1, and F2 isolates. Contiguously, nucleotide sequence TTTAGTCAGTG (nt + 154 to + 164) which includes the AP-1 (III) binding site was conserved in CRF12_BF (80%) as well as in subtypes F2 (80%), B (65%), D (40%) and A1 (10%) isolates. For this TFBS, specific variants such as TCTAGACTGAGT was found in CRF22_01A1 and CRF01_AE, whereas in subtype C and CRF02_AG specimens, respectively, the consensus TT(G/T)TGGT(A/G)GTG and T(A/C)TAGA(C/T)TGTG were observed (Fig. 2). The adjoining TGGAAAATCTCTA sequence (nt + 165 to + 177) carrying the recognition site for AP3-L /NF-AT^27,31^, displayed a mutation of the two Gs (one deletion and one substitution to an A) in most of the U5 sequences from subtype A1 and G isolates, as well as in their related CRFs (Fig. 2). The resulting TAAAAATCTCTA variant modified the overlapping sequence TCAGTGTGGAAAATC that has been reported as a binding site for C/EBP proteins^29^.

### Strain differential variability in the nucleotide sequence spanning the PBS

In this region, the 18 nucleotides sequence (nt + 182 to + 199) corresponding at the RNA level to the PBS, was entirely conserved in our study (Fig. 2). The adjacent nucleotides (nt + 200 to + 214) was fitting into the consensus sequence NNGAAAG(C/T)GAAAGDD in all HIV-1 strains studied except in CRF02_AG. This consensus, closely related to the interferon-stimulated response element (ISRE), was shown to interact with members of the interferon responsive factor (IRF) family^27,32–34^. The 7 nucleotide deletion observed at this position in CRF02_AG specimens was concordant with a previous report^26^. Its resulted in an ACCGGAAG(C/T/A) consensus (Fig. 2), that includes a perfect match to the high affinity binding domain reported for several members of the ETS family of transcription factors^35^.

Contiguous to the IRF motif, an insert of about 24nt was found in sequences belonging to subtype G, CRF01_AE, and CRF02_AG isolates as previously reported^24,26,36^. In our study, the 24nt insert was also displayed by the CRF22_01A1 specimens, and by 20% of the subtype A1 sequence. Interestingly, this insert seems to restore an ISRE-like element in the CRF02_AG, while it was duplicated in the other strains (Fig. 2). Downstream, the overlapping nucleotide matched the GDDRAACCRGAGRAGM consensus (nt + 212 to + 227), in CRF12_BF (100%) as well as in subtypes F2 (100%), D (90%), B (80%), C (12.5%) and A1 (10%). In contrast, its variant GDDRGACCAGAGRAGA was specific to subtype C specimens. Due to a 3nt deletion in specimens of subtypes A1, CRF02_AG, CRF01_AE, and CRF22_01A1 (Fig. 2), we observed that this sequence was converted to a GTTCCAGAGAAG(A/C/T) consensus. This consensus includes a TTCCAGAGAA motif described as the preferential binding site for STAT6 and a weak binding site for STAT5^37,38^. Further downstream, point mutations in the sequence overlapping the potential TFBS for STAT6 proteins (nt + 225 to + 229) generated a potential recognition site for the GATA family of proteins (Fig. 2).

**Figure 4:**
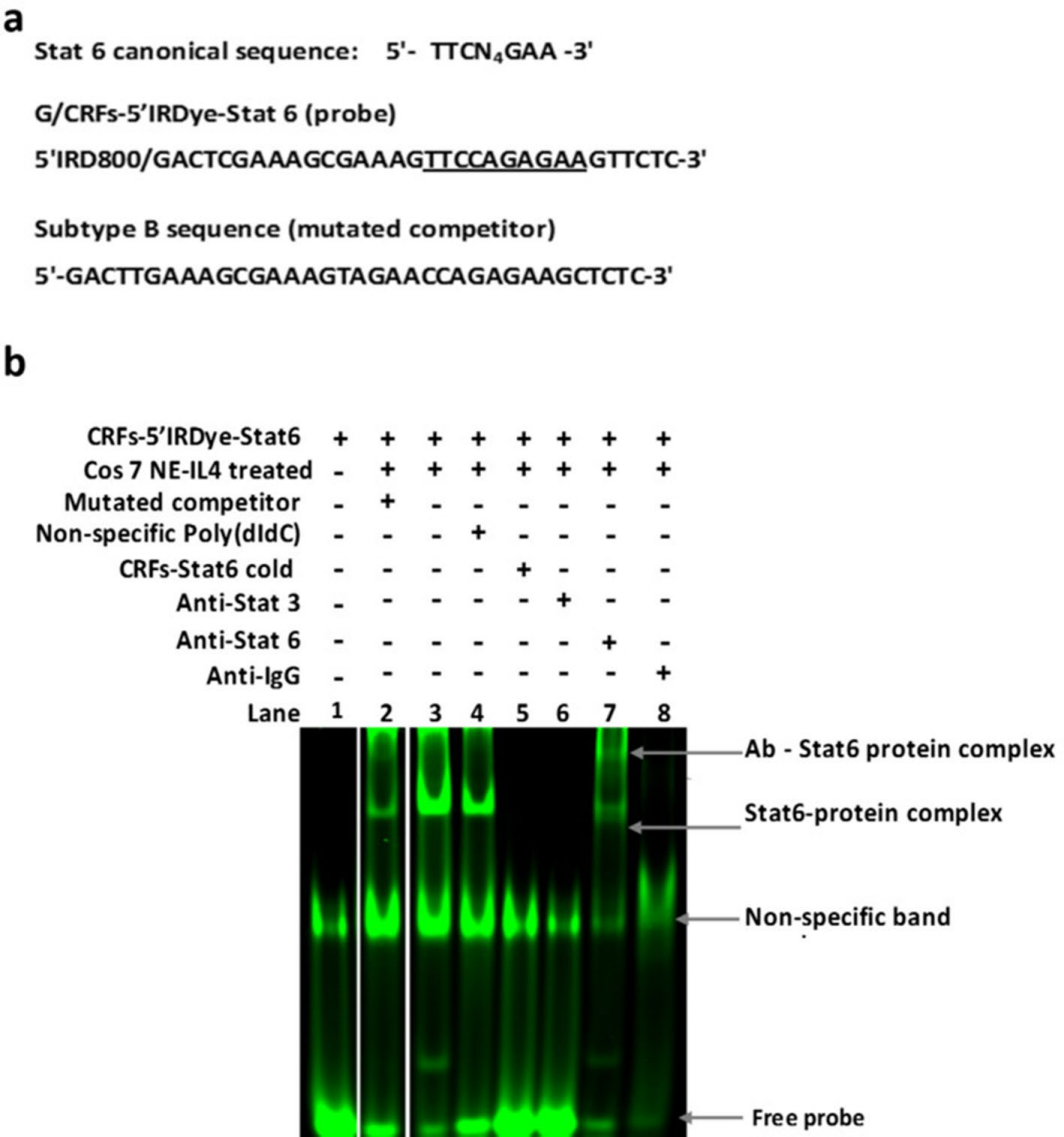
Strain-specific sequence motif for the binding site for STAT6 transcription factor in the PBS region of clade A, G, and related CRFs (02_AG, 01_AE and 22_01A1). (**a**) Sequences from nucleotide + 197 to + 232 used for the fEMSA. The G/CRFs-5′IRDye-STAT6 sequence from Subtype G and non 12_BF CRFs used as a probe, includes the reported STAT6 canonical motif. Its equivalent from subtype B was used as a mutated competitor. (**b**) The CRFs-5′IRDye-STAT6 probe (lane 1) was incubated in the absence (lane 1) or with 5 µg of nuclear extract from IL-4 stimulated Cos 7 cells (Lanes 2–7). In this gel-shift experiment, we noted the presence of a non-specific band even in the absence of nuclear extract (lanes 1–8). This suggests that compounds enhancing binding conditions in this experiment might bind to the designed oligonucleotide. More importantly, we observed a specific band for the DNA probe-protein complex (lane 3). The specificity of STAT6 protein and DNA probe binding was shown by the non-disruption of the protein-DNA complex in the presence of a 200-fold excess of the mutated competitor (lane 2) or non-specific oligonucleotide (poly(dI-dC), lane 4) and by the disruption of the DNA–protein complex in the presence of 100-fold excess of the cold probe (CRFs-STAT6, lane 5). Additionally, we observed a supershift of the DNA–protein complex with anti-STAT6 antibody (lane 7), and not with anti-STAT 3 (lane 6) or non-specific IgG (lane 8). Surprisingly, no shift was detected with anti-Stat3 and anti-IgG antibodies (lanes 6 and 8). This might suggest that in these specific conditions, the presence of these later antibodies may disrupt the protein–DNA interaction, resulting in loss of the Stat6-DNA complex. To improve the conciseness and clarity, the image was cropped and the bands of interest that were non-adjacent in the original gel (Supplementary information S1 Fig. 4), were juxtaposed with a clear separation.

Evidence of a strain-specific binding site for STAT6 factor at this position was shown by fEMSA using sequence derived from subtype G and non 12_BF CRFs used as a probe (Fig. 4).

### Strain differential nucleotide variability in the GLS sequence

In the GLS sequence, nucleotide sequence GACGCAGGACTCG (nt + 234 to + 246) in which we observed an Ets-1-like core motif (CAGGA), was conserved in almost all our specimens. Only CRF02_AG sequences displayed some mutations, such as a 1nt insertion, as previously reported^26^. In our study, this insert was also observed in a few CRF22_01A1 and subtype A1 specimens (Fig. 2). The strain-specificity that we found in the motif corresponding to the DIS loop was concordant with previous reports^36,39^. Indeed, the GCGCGC motif (nt + 257 to + 262) displayed in subtypes B and D isolates was changed to a GTGCAC variant in all other strains including CRF22_01A1 (Fig. 2).

**Figure 5:**
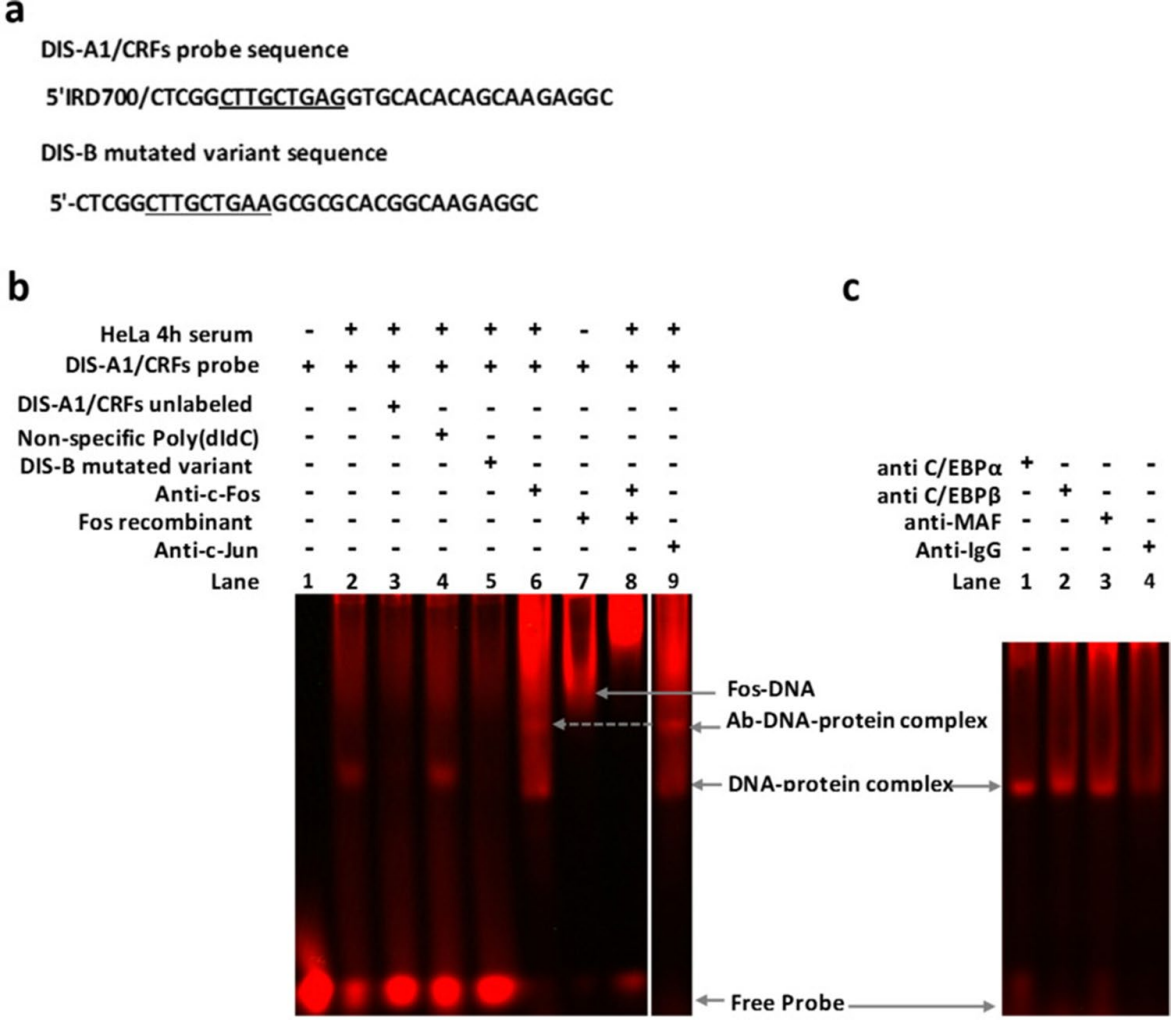
EMSA analysis of the sequence spanning the DIS motif predicted to include binding sites for C/EBP and AP-1 factors. (**a**) Sequences from nucleotides + 243 to + 274 in the GLS region. The sequence from subtype A1 and related CRFs as a probe (DIS-A1/CRFs). The sequence used as a mutated competitor (DIS_B) includes a perfect match for the C/EBP canonical motif. DIS-A1/CRFs was incubated in the absence of nuclear proteins (**b** lane 1) or with 5 µg of HeLa 4-h serum response nuclear extract (**b** lanes 2–9 and **c** lane 1–4). (**b**) The observed DNA–protein complex (lane 2) was displaced by the cold probe (lane 3) and by the mutated competitor (lane 5), indicating a positive competition. In contrast, there was no disruption of the DNA–protein complex in the presence of a non-specific competitor (lane 4). The presence of AP-1 proteins within the DNA-proteins complex was indicated by the binding of recombinant c-Fos protein (lane 7) and the supershift reaction with anti c-Fos (lane 6) and c-Jun (lane 9) antibodies. (**c**) In a continuation gel run in parallel, we observed the lack of a supershift in the presence of anti-C/EBP α (lane 1) and β (lane 2) antibodies. This suggests the absence of binding sites for these proteins on the oligonucleotide used as a probe. Also, we noted that the amount of probe used was significantly consumed in the reaction when compounds such as recombinant protein and/or antibodies were added. However, no band shift was formed between the probe and the antibodies alone (**b** lanes 6–9, **c** lanes 1–4 and supplementary information S1 Fig. 5). This could suggest an interaction between AP-1 related antibodies and the probe. However, in this experiment no complex was formed between the probe and the antibody alone (supplementary information S1 Fig. 5). To improve the conciseness and clarity, the image was cropped and the bands of interest that were non-adjacent in the original gel (Supplementary information S1 Fig. 5) were juxtaposed with a clear separation.

In the sequence spanning the DIS loop, we noticed that variation of the A255, A256, and A263 nucleotides (AA; A) resulted in a few strain-specific patterns such as AA; T in subtype C and AG; (A/G) in a majority of sequences belonging to subtypes A1 and G specimens and their related CRFs (Fig. 2). Interestingly, the A255A256 patterns create along with their contiguous nucleotides (+ 248 to + 254), a CTTGC(T/C)GAA consensus that perfectly matches the canonical motif (C/A)TTNCNN(C/A), reported as the best fit for the binding of C/EBP proteins^40^. In-silico analysis of this sequence predicted a binding site for C/EBP proteins as well as and c-Jun and c-Fos factors. However, fEMSA analysis revealed a binding site for c-Fos and c-Jun,but not for C/EBP proteins in sequence from subtype A1 and related CRFs (Fig. 5).

Inter and intra-strain variability at the 3′ end of the sequence spanning the DIS hairpin were limited to single point mutations. Hence, a G265A mutation was found in a majority of non-B and C subtype sequences. This latter mutation seemed to convert the wobble 253U-G265 reported in the DIS hairpin^20^, into a typical 253U-A265 Watson and Crick base pairing. The contiguous nucleotide sequence reported to include binding sites for Sp 1 family of proteins, also showed some strain-specific variability^27^. Thus, the Sp 1(I) motif GAGGCGAGG (nt + 270 to + 278) was mutated to GAGGCGAGA in a majority of sequences belonging to strains other than subtypes B and D. Sp 1(II) (nt + 279 to + 289), which overlaps the 5′ end of the sequence corresponding to the SD hairpin, displayed greater variability with some strain-specific patterns such as GCAGCGRACG observed in subtype D isolates (Fig. 2). In the Sp 1 (II) variant of subtype D, mutations mostly occurred within the central GCGG motif, important for the recruitment of the Sp 1 and related proteins^41^. Furthermore, we observed that variation in this motif and surrounding nucleotides might modify base paring described in the secondary structure of the SD hairpin (G282A-C300T, C287A-G294, and T288C-A293), in subtype D isolates.

**Figure 6:**
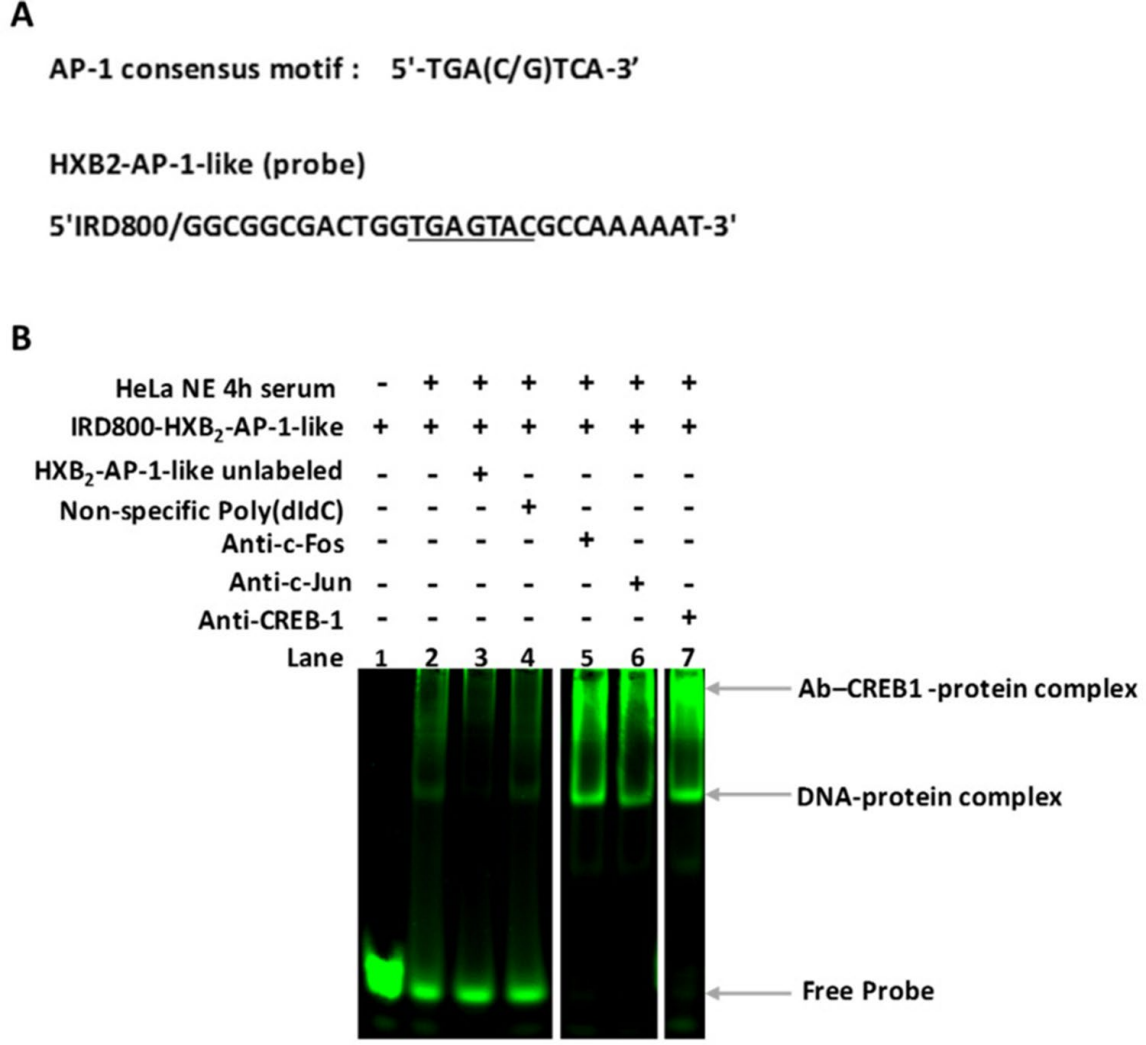
EMSA analysis of the GLS sequence, including an AP-1-like motif at the 3′end of the SD domain. (**a**) The conserved oligonucleotide spanning the SD loop motif predicted to include an AP-1-like motif was used as an IR labeled probe. (**b**) 4-h serum response Hela Nuclear extract (Active Motif) was incubated with antibodies against c-Jun, c-Fos, or CREB-1 antibodies at 4 °C for 24 h before the addition of the HXB2-AP-1-like (probe). A band representing the complex antibody-DNA–protein supershift was observed in the presence of anti -CREB-1 antibodies only (lane 7), and not with anti c-Fos (lane 5) or c-Jun (lane 6). The presence of the protein-DNA complex (lane 2) was abolished by the addition of an excess of the cold probe (lane 3) and not disrupted by the non-specific poly (dI-dC) competitor (lane 4) indicating the specificity of the DNA–protein complex. The amount of probe used in this experiment seemed to be significantly consumed in the reaction when additional compounds such as antibodies were added. However, no shift was formed between the probe and the antibodies alone (Supplementary information S1 Fig. 6). To improve the conciseness and clarity, the image was cropped and the bands of interest that were non-adjacent in the original gel (Supplementary information S1 Fig. 6), were juxtaposed with a clear separation.

Unlike the 5′SD stem, the tetramer motif corresponding to the SD loop reported as the major splice donor^17^, was conserved in our study. The nucleotide sequence overlapping the 3′ end of this motif (nt + 290 to + 299) was also well conserved in all the HIV-1 strains studied. This sequence includes a TGAGTAC motif closely related to the canonical AP-1 binding site (TGAG/CTCA) with a CA to AC transversion (Fig. 6a). Supershift analysis of a sequence containing this AP-1- like motif showed a DNA–protein complex that was displaced by antibodies against CREB-1 proteins (Fig. 6b). No supershift was observed with antibodies against c-Jun and c-Fos, proteins (Fig. 6b).

The subsequent sequence, including the motif corresponding to the packaging signal (PSI), was highly conserved in most sequences analyzed. However, we noticed a G317A mutation that converted the consensus GGAG into an AGAG motif in the F2 and CRF12_BF GLS mainly (Fig. 2).

## Discussion

In this study, we described the diversity of the U5, PBS, and GLS regions of the 5′ end of genetically diverse HIV-1 strains. Due to its critical role in the life cycle of HIV-1, this section of the viral genome has been thought to be highly conserved. However, with an average of 17% of nucleotide changes, we found that the inter-strain variability of the U5 and downstream sequence was in the same range as *gag* and *env* genes^42^. Subtype A1 was the only variant to display significant intra-subtype variability. This was mainly due to the presence of a 24nt insertion and a 3nt deletion in the PBS region of only one-fifth of the sequences from subtype A1 isolates, while this same mutational profile was observed in all sequences from subtype G, CRF02_AG, CRF01_AE isolates as previously described^24,43,44^, and also in all CRF22_01A1. Intra-strain diversity of subtype A1 sequence may be explained by the fact that it belongs to one of the most divergent variants of HIV-1 group M. Indeed, HIV-1 subtype A contains at least six sub-subtypes and may probably include sequences resulting from intra-subtype recombination^45,46^. This hypothesis is also supported by the fact that although the global prevalence of HIV-1 subtype A is considerably lower than subtype B, more than 50% of CRFs reported in the Los Alamos Database contain subtype A-attributed genome fragments.

Footprint and gel shift experiments have allowed the identification of several TFBS on the HIV-1 promoter. Nonetheless, there may be other sites yet to be identified, especially in the U5 region and downstream sequence. In the U5 region, the binding of various factors such as AP-1 and NF-AT have been reported to play a positive role on HIV-1 transcription and replication^27,29,47^. Here, we observed that AP-1 (II) in CRF01_AE and CRF22_01A1 specimens, diverged from its consensus sequence. This variation could potentially result in a differential influence of this TFBS on the replication of these HIV-1 CRFs, as it was demonstrated that that AP-1 (II) is critical for HIV-1 transcription and replication^27^. AP-1 factors are homo and heterodimers of proteins from Jun, Fos, ATF/CREB, and Maf families capable of recognizing the same or similar binding motifs. Although we did not explore its binding specificities, variability in AP-1 (III) sequence suggests its distinctive ability to modulate HIV-1 replication and expression. Characterization of this TFBS has suggested that it could mediate HIV-1 gene expression by interacting with transcription factors composed of components other than, or in addition to the AP-l /Jun/Fos^27,48^. Also, AP-1 (III) may not affect the binding of AP-1 related factors but of the overlapping High-Mobility-Group Protein I, which modulates the binding of transcription factors to the HIV-1 promoter^48^.

In the GLS region, we identified novel potential targets for the binding of c-Jun, c-Fos proteins in the sequence spanning the dimerization site of subtype A1 related CRFs. Also, similar to what has been reported in the LTR of the Moloney Leukemia retrovirus^49^, we showed that mutation involving a 2-base pair transversion of the canonical AP-1 binding site does not allow the binding AP-1 factor components such as c-Fos and c-Jun. The binding of CREB-1 factors in this AP-1 like motif may be explained by the close similarity between binding sites for AP-1 and CREB/ATF family of transcription factors.

Small differences in the consensus of a TFBS can dictate the binding specificity of a factor and its subsequent action on genome activity. For members of the bHLH family, discrimination in the binding is determined by the two-central dinucleotide of their canonical core motif CANNTG and by the flanking nucleotides^50,51^. Thus, binding of distinctive bHLH on specific E-box motifs described along the HIV-1 5′LTR has been reported to modulate viral replication and the establishment of latency^52,53^. Here, we identified a potential E-box (5′-CATCTG-3′) in the U5 region of all CRF22_01A1 specimens. This E-box motif differs from the preferred binding site for USF-1^54^, and therefore, it was surprising to observe the binding of this factor on a 5′-CATCTG-3′ motif. Nevertheless, our finding was in line with previous reports demonstrating the binding of USF-1 to non-canonical motifs^55^, and its tolerance to nucleotide variation^50^. On the other hand, Tal1 seems to demonstrate a lower binding affinity 5′-CATCTG-3′ motif, despite its similarity to a reported Tal1 binding motif^56^. E-boxes have been mainly identified in the U3-R region of the 5′HIV-1 LTR. To the best of our knowledge, this is the first evidence of the presence of an E-box within the U5 region of the HIV-1 genome. This might be explained by the fact that most studies identifying putative TFBS in this region did not evaluate divergent HIV-1 variants.

**Figure 7:**
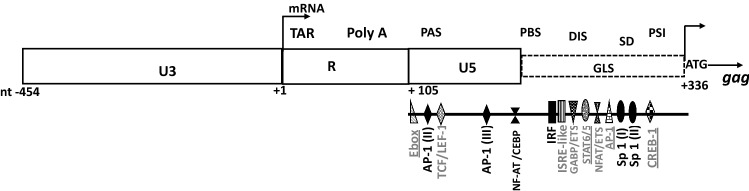
Summary of the location of known (black) and newly identified (grey) potential transcription factor binding sites reported in the sequence studied. Potential TFBS revealed by gel shift are assays are underlined.

IRF-1 by interacting with ISRE was shown to initiate HIV-1 transcription in the absence of Tat and to amplify its replication cooperatively with Tat^47^, whereas the deletion of the ISRE motif reduced the replicative capability of the virus^57^. Here, we report a potential duplication of the ISRE-like motif in the PBS region of CRF01_AE, CRF22_01A1, and subtype G specimens. The presence of a supplementary ISRE suggests that these strains could demonstrate additional capabilities to modulate transcription and replication. This hypothesis seems reasonable since it was shown that an HIV-1 strain could gain a selective advantage with the acquisition of transcription factor-binding sites. This was reported for HIV-1 subtype C with the additional binding site for NF-kB, and for CRF01_AE with the conversion of one NF-kB into a GABP binding site in the enhancer region of the 5′LTR^58,59^. Moreover, the proximity to the ISRE of binding sites for STAT6 as well as ETS factors as predicted in CRF02_AG specimens, may favor a cooperative interaction of these transcription factors in a strain-specific manner. Indeed, ISRE interacts with both IRFs and STAT proteins as multi-protein DNA-binding complexes that may cooperate with co-acting transcription factors such as NF-κB and PU.1^60^. The newly identified TFBS discussed in this manuscript, along with those previously described in this region of the 5′ end of the HIV-1 genome are summarized in Fig. 7.

The high conservation of the motifs corresponding to the PAS, PBS, DIS, and SD domains in the 5′ UTR suggests their functions might be irrespective of the HIV-1 strain. Nonetheless, the integrity of the structure of the 5′ UTR has been suggested to be critical for its optimal functionality^21^. In this study, we report strain-specific mutations within flanking nucleotides that could impact classical base pairing resulting in a potential rearrangement of the secondary structure in some HIV-1 strains such as subtype D. This rearrangement could lead to a strain distinctive modulation by the 5′ UTR of several steps of the life cycle of HIV-1. In HIV-1 MAL isolates, for example, it was demonstrated that an extensive interaction of the viral RNA with tRNA_3_^Lys^ is required for efficient initiation of reverse transcription, while in HXB2/ NL4.3, the structure of the vRNA/tRNA_3_^Lys^ complex is minimally affected^61^. Also, in the case of HIV-1 MAL isolates, transition between initiation and elongation of the reverse transcription was facilitated compared to Lai/HXB2/NL4-isolate^62^. These observations can be extended to HIV-1 subtype G, CRF02_AG, and CRF01_AE reported to cluster with MAL isolates^63^. According to our findings, CRF22_01A1 strains may be likely considered as MAL-like with closely related biological properties. Also, as DIS flanking nucleotides were shown to affect RNA association rates^63^, our results suggest that their variability might differentially impact the dimerization process of diverse HIV-1 strains.

## Conclusion

In conclusion, we observed several strain-specific mutations in the U5-GLS region of genetically diverse HIV-1 strains analyzed in our study. This variability could potentially affect the replication fitness of HIV-1 through differential interaction with strain-specific TFBS and/or rearrangement in the 5′ UTR secondary/tertiary structure. Future studies involving DNA–protein interactions might provide useful information on the role of specific TFBS variation on viral replication. Better knowledge of the variability of this critical region of the HIV-1 genome may provide new insights into the molecular mechanism underlying various steps of the life cycle of HIV-1 and the observed differences in biological properties of divergent HIV-1 strains. The absence of functional investigations was a limitation of this study. Further investigations on the potential TFBS newly identified in our study and to the strain-specific secondary structures of this region of the HIV-1 genome are warranted.

## Materials and methods

### Specimens, RNA isolation and sequencing

This study was done with a total of 80 contigs obtained from a previously described methodology^1^. Briefly, RNA was extracted from selected plasma derived viruses belonging to subtypes B, C, D, F2 and G, as well as CRF02_AG, CRF01_AE and CRF22_01A1 (Table 1). Total RNA was reverse transcribed using Superscript III First-strand synthesis System for RT-PCR (Invitrogen 18080-051). The cDNA was amplified by polymerase chain reaction (PCR). Expected PCR products of about 850 base pairs were gel purified using QIAquick Gel Extraction Kit (QIAGEN, Hilden, Germany). Deep sequencing of the purified PCR products was carried out at the core facility of the Food and Drug Administration (FDA, White Oak, MD, USA). Fastq files generated by Illumina sequencing were processed with CLC Genomics Workbench version 9.0 (CLC bio/Qiagen, Aarhus, Denmark). As previously reported, the subtype assigned to the 80 retrieved contigs is summarized in Table 1.

**Table 1:** Specimen description and LTR subtype assignment.

County of origin	Primary genotype	LTR subtype									Total
		B	C	D	F2	12_BF	A1	G	02_AG	01_AE	
USA, France, Spain, Japan, China, Venezuela, Bolivia	B	20				5					25
South Africa, Malawi, India	C		8								8
Uganda, Cameroon	D			10							10
Spain	F1				1						1
Cameroon	F2				3						3
Rwanda, Kenya, Uganda, Russia, Spain, Tanzania	A1						10				10
Cameroon, Kenya, Spain	G							3			3
Cameroon, Kenya, Spain	02_AG				1				9		10
China	01_AE									5	5
Cameroon	22_01A1									5	5

B = Subtype B; C = Subtype C; D = Subtype D; F1 = Subtype F1; F2 = Subtype F2; A1 = Subtype A1; G = Subtype G; BF = CRF12_BF; AG = CRF02_AG; AE = CRF01_AE; 01A1 = CRF22_01A1.

### Nucleotide sequence analysis

Sequences from the same subtypes were aligned in MEGA 7 using the 5′ end of HXB2 sequence (accession number K03455) as a reference to position nucleotide from the U5 region to upstream of the *gag* start codon. Mutations were identified by comparing sequences from each HIV-1 strain to the reference HXB2, and within matched subtype or CRF. Identification of potential regulatory elements was based on similarity with reported consensus motifs, prediction in silico with the ConTra v3 webserver^64^ (https://bioit.dmbr.ugent.be/ConTra/index.php) or the Match—1.0 Public program (https://gene-regulation.com/pub/programs.html). A pairwise comparison in CLC Genomics Workbench was used to calculate the percentage of identity.

### Infrared fluorescent electrophoretic mobility shift assay (fEMSA)

It is well known that there is no single set of binding and electrophoresis conditions that work well for all molecular systems. Thus, to analyze the ability of the predicted HIV-1 strain specific DNA motifs to bind their respective transcription factors, fEMSA experiments were optimized by assessing several binding and electrophoresis conditions as well as selected nuclear extracts known to express the DNA binding proteins of interest. For this reason, Jurkat, Cos-7, and Hela nuclear extracts were selected to study potential binding motifs for basic helix-loop-helix (bHLH), STAT, and AP-1 related factors, respectively. HeLa (4 h. serum response) and Jurkat nuclear extracts were purchased from Active Motif (Carlsbad, CA). Cos-7 cells were grown in Dulbecco’s modified Eagle medium (Invitrogen) containing 10% fetal calf serum, 1% penicillin/streptomycin, and 2 mM glutamine. After 3 h of treatment with 20 ng/ml of recombinant Human IL-4 (Sigma), cells were collected, and nuclear proteins were extracted using Nuclear Extraction Kit (Abnova, Tapei, Taiwan) according to the manufacturer’s instructions.

EMSA with Infrared labeled oligonucleotides was adapted from a previously published protocol^65^. Briefly, 5 µg of nuclear extract proteins or 1–3 µg of recombinant protein, was incubated with 0.1 µM of a double-stranded infrared Fluorescent Dye-labeled probe for 25 min at room temperature in a 16 µl binding reaction containing 10% (v/v) glycerol, 20 mM HEPES (pH 7.6), 50 mM KCl, 10 mM NaCl, 5 mM MgCl_2_, 1 mM EDTA, 1 mM DTT, 10 μg of BSA (GE Amersham Biosciences, UK), 50 ng of poly (dI-dC) and 0.01% NP-40 (Invitrogen, Carlsbad, CA). Also, 4 mM urea, 0.1 mM ATP and 20 mM spermine were added in the binding reaction with nuclear extract from IL-4 stimulated Cos-7 cells. Optionally (when indicated), a 200-fold molar excess of cold probe or 100-fold molar excess of unlabeled mutant or 1ug of poly (dI-dC) was added as a specific, mutated or non-specific competitor respectively.

For supershift assays, 2 to 5 µg of antibodies against several different antibodies including c-Fos (sc-8047X), c-Jun (sc-376488X), Fos B (sc-398595 X), Fra-1 (sc-28310 X), MafF/G/K (sc-166548X), CREB-1 (sc-377154X), ATF-1 (sc-270X), C/EBP α (sc-166258 X), C/EBP β (sc-7962X), USF-1 (sc-390027X), Tal1 (61,260, Active motif), c-Myc (61,076, Active motif), AP-4 (sc-166024X) , STAT3 (sc-8019X), STAT6 (sc-374021X) or a purified mouse immunoglobulin (sc-2025) was added in an intermediate incubation prior to the addition of the labeled probe. 2 μL of 10 × Orange G loading dye (LI-COR Biosciences Inc., Lincoln, NE) supplemented with 10% glycerol (v/v) were then added to each binding reaction before electrophoresis on 4% polyacrylamide gels in 0.5× TBE at 80 V for 2 h at 4 °C. The gel was scanned directly in the glass plates with the Odyssey CLx imaging system (LI-COR Biotechnology Division, Lincoln, NE). The oligonucleotides designed for this study were synthesized, annealed, infrared labeled, and purified by Integrated DNA Technologies, Inc (IDT).

### Disclaimer

The findings and conclusions in this article have not been formally disseminated by the Food and Drug Administration and should not be construed to represent any Agency determination or policy.

## Supplementary information

Supplementary Information 1.

## Data Availability

The data for this study is available from the corresponding author on request in compliance with the FDA policy.
