# Supplementary material for: Genetic variability of the U5 and downstream sequence of major HIV-1 subtypes and circulating recombinant forms
Source: Sci Rep. 2020 Aug 6;10:13214. doi: 10.1038/s41598-020-70083-1 (PMC7411029; doi:10.1038/s41598-020-70083-1)

Genetic variability of the U5 and downstream sequence of major HIV-1 subtypes and circulating recombinant forms.

Christelle Mbondji-wonje [1] *, Ming Dong [2, 3], Jiangqin Zhao [1], Xue Wang [1], Aubin Nanfack [4] Viswanath Ragupathy [1], Ana M. Sanchez [5], Thomas N. Denny [5] and Indira Hewlett [1*]

Original images of the gels used for Figure 3

A

**Jurkat Nuclear extract**	-	+	+	+	+	+	-	+	-	+
**CRF22-5’IRDDye-Ebox**	+	+	+	+	+	+	+	+	+	+
**CRF22-Ebox cold**	-	-	+	-	-	-	-	-	-	-
**Non-specific Poly(dIdC)**	-	-	-	+	-	-	-	-	-	-
**Clade B mutant**	-	-	-	-	+	-	-	-	-	-
**Anti-AP4**	-	-	-	-	-	+	-	-	-	-
**Anti-Tal1**	-	-	-	-	-	-	+	+	-	-
**Anti-USF1**	-	-	-	-	-	-	-	-	+	+
**Lane**	**1**	**2**	**3**	**4**	**5**	**6**	**7**	**8**	**9**	**10**

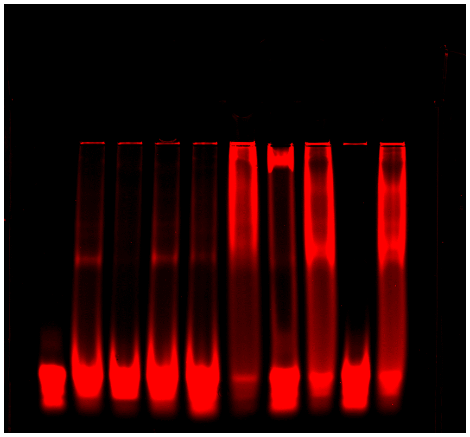

B

**Jurkat Nuclear extract**	-	-	-	-	+	+	+	+	+	+	+	+
**CRF22-5’IRDDye-Ebox**	+	+	+	+	+	+	+	+	+	+	+	+
**anti-cMyc**	-	-	-	-	+	-	-	-	-	+	-	-
**anti-TCF3**	-	-	-	+	-	-	-	-	+	-	-	-
**anti-Gata3**	-	-	+	-	-	-	-	+	-	-	-	-
**anti-Gata2**	-	+	-	-	-	-	+	-	-	-	-	-
**anti-Gata1**	-	-	-	-	-	+	-	-	-	-	-	-
**Anti-IgG**	-	-	-	-	-	-	-	-	-	-	+	-
**Lane**	**1**	**2**	**3**	**4**	**5**	**6**	**7**	**8**	**9**	**10**	**11**	**12**

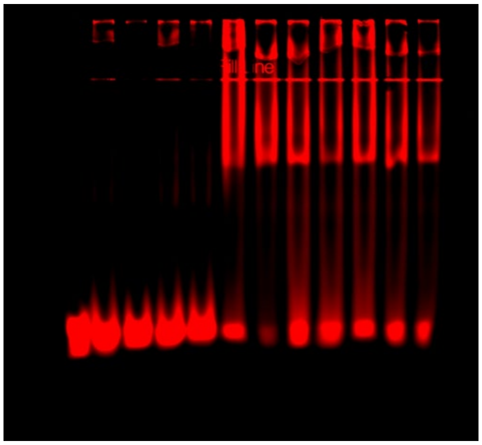

Original image of the gel used for Figure 4

IRD800-HXB$_2$-AP-1-like	+	+	+	+	+	+	+	+	+	+	+	+
Cos 7 NE-IL4 treated	-	+	+	+	-	-	+	+	+	+	+	+
Mutated competitor	-	-	-	+	-	-	-	-	-	-	-	-
STAT6 recombinant	-	-	-	-	+	+	-	-	-	-	-	-
Non-specific Poly(dIdC)	-	-	-	-	-	-	-	+	-	-	-	-
CRFs-Stat6 cold	-	-	-	-	-	-	-	-	+	-	-	-
Anti-Stat 3	-	-	-	-	-	-	-	-	-	+	-	-
Anti-Stat 6	-	-	-	-	-	-	-	-	-	-	+	-
Anti-IgG	-	-	-	-	-	-	-	-	-	-	-	+
Lane	1	2	3	4	5	6	7	8	9	10	11	12

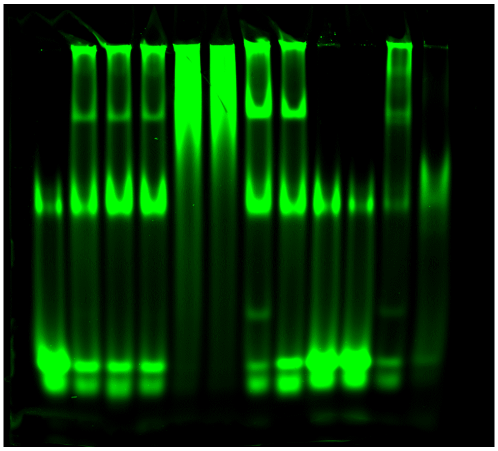

Original images of the gels used for Figure 5

A

HeLa 4h serum	-	+	+	+	+	+	-	-	+	+	-	-	-	+	+
DIS-A1/CRFs probe	+	+	+	+	+	+	+	+	+	+	+	+	+	+	+
DIS-A1/CRFs unlabeled	-	-	+	-	-	-	-	-	-	-	-	-	-	-	-
Non-specific Poly(dIdC)	-	-	-	+	-	-	-	-	-	-	-	-	-	-	-
DIS-B mutated variant	-	-	-	-	+	-	-	-	-	-	-	-	-	-	-
Anti-c-Fos (5ug)	-	-	-	-	-	+	-	+	-	-	-	-	-	-	-
Fos recombinant	-	-	-	-	-	-	+	+	-	-	-	-	-	-	-
Anti-c-Jun (c-376488X), 5ug	-	-	-	-	-	-	-	-	-	+	-	-	+	-	-
Anti-c-Jun (active motif), 5 ug	-	-	-	-	-	-	-	-	+	-	-	+	-	-	-
Jun recombinant	-	-	-	-	-	-	-	-	-	-	+	+	+	-	-
Anti-c-Jun (2 ug)	-	-	-	-	-	-	-	-	-	-	-	-	-	+	-
Anti-c-Fos (2 ug)	-	-	-	-	-	`	-	-	-	-	-	-	-	-	+
Lane	1	2	3	4	5	6	7	8	9	10	11	12	13	14	15

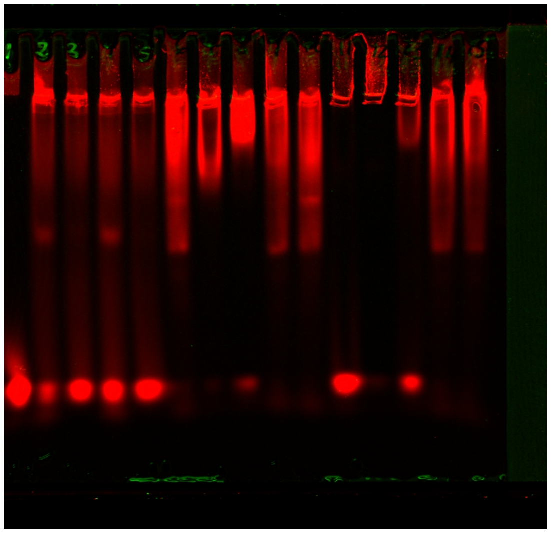

B

HeLa 4h serum	-	+	+	+	-	-	-	+	+	+	+	+	+	+	+
DIS-A1/CRFs probe	+	+	+	+	+	+	+	+	+	+	+	+	+	+	+
DIS-A1/CRFs unlabeled	-	-	+	-	-	-	-	-	-	-	-	-	-	-	-
Non-specific Poly(dIdC)	-	-	-	+	-	-	-	-	-	-	-	-	-	-	-
Fos recombinant	-	-	-	-	+	-	-	-	-	-	-	-	-	-	-
CREBBP protein	-	-	-	-	-	+	-	-	-	-	-	-	-	-	-
Jun recombinant	-	-	-	-	-	-	+	-	-	-	-	-	-	-	-
Anti-c-Jun (c-376488X), 5ug	-	-	-	-	-	-	-	+	-	-	-	-	-	-	-
Anti-c-Jun (2 ug)	-	-	-	-	-	-	-	-	+	-	-	-	-	-	-
anti C/EBPβ	-	-	-	-	-	-	-	-	-	+	-	-	-	-	-
anti C/EBPα	-	-	-	-	-	-	-	-	-	-	+	-	-	-	-
anti-MAF	-	-	-	-	-	`	-	-	-	-	-	+	-	-	-
anti-IgG	-	-	-	-	-	`	-	-	-	-	-	-	+	-	-
anti-ATF	-	-	-	-	-	`	-	-	-	-	-	-	-	+	-
anti-FRA	-	-	-	-	-	`	-	-	-	-	-	-	-	-	+
Lane	1	2	3	4	5	6	7	8	9	10	11	12	13	14	15

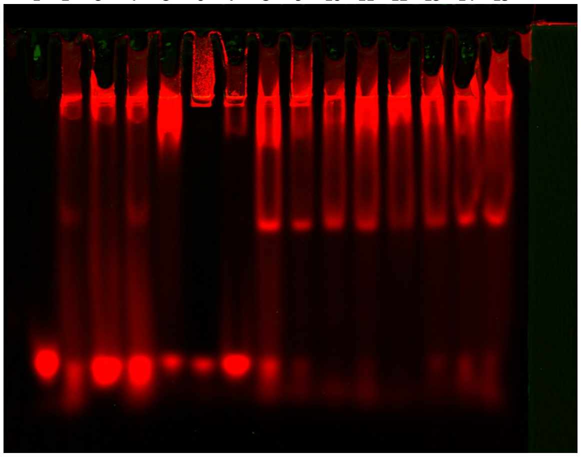

Original image of the gel used for figure 6

**HeLa NE 4h serum**	-	+	+	+	+	+	-	-	-	+	+	+
**IRD800-HXB_2-AP-1-like**	+	+	+	+	+	+	+	+	+	+	+	+
**HXB_2-AP-1-like unlabeled**	-	-	+	-	-	-	-	-	-	-	-	-
**Non-specific Poly(dIdC)**	-	-	-	+	-	-	-	-	-	-	-	-
**CREB-1 Antibody(24H4B)X**	-	-	-	-	+	-	-	-	-	-	-	-
**CREB-1 (sc-377154X_D12)**	-	-	-	-	-	+	-	-	-	-	-	-
**CREBBP recombinant**	-	-	-	-	-	-	+	-	-	-	-	-
**CREB1 recombinant + anti _D12)**	-	-	-	-	-	-	-	+	-	-	-	-
**CREB1 recombinant + anti 24H4B)**	-	-	-	-	-	-	-	-	+	-	-	-
**Anti-c-Fos**	-	-	-	-	-	-	-	-	-	+	-	-
**Anti-c-Jun**	-	-	-	-	-	-	-	-	-	-	+	-
**Fos recombinant**	-	-	-	-	-	-	-	-	-	-	-	-
**Lane**	**1**	**2**	**3**	**4**	**5**	**6**	**7**	**8**	**9**	**10**	**11**	**12**

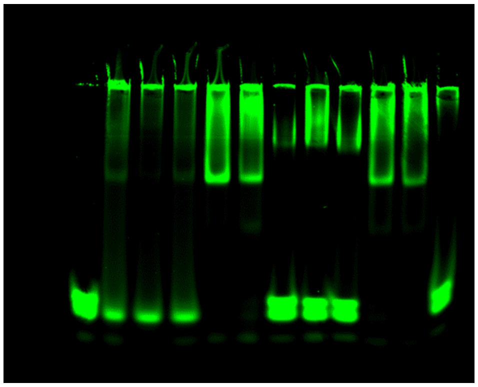

Supplement: Supplementary file 1 — Supplementary Information 1. [file 41598_2020_70083_MOESM1_ESM.pdf]
